# 胸腺肿瘤专题前言

**DOI:** 10.3779/j.issn.1009-3419.2014.02.02

**Published:** 2014-02-20

**Authors:** 克能 陈, 文涛 方

**Affiliations:** 1 100142 北京，北京大学附属肿瘤医院胸外科 Department of Thoracic Surgery, Peking University Cancer Hospital, Beijing 100142, China; 2 200030 上海，上海交通大学附属胸科医院胸外科 Department of Thoracic Surgery, Shanghai Chest Hospital, Shanghai Jiao Tong University, Shanghai 200030, China

胸腺肿瘤是一类少见疾病，发病仅为0.17/10万，亚裔人种略高于白种人，约0.3-0.4/10万。胸腺瘤研究与投入均较少，诊断与治疗缺乏统一的指南，尤其在术前定性诊断、组织学分型、术前治疗、手术方式、术后治疗以及对术后复发的治疗等一系列问题上均处于不规范状态。很多医生也对该疾病的治疗经验有限，而且研究进展缓慢，但是对于患者和家属，这是不可接受的理由。2003年美国患者Barbara Neibauer身患胸腺肿瘤，虽经积极的治疗，但仍于两年后去世。Barbara Neibauer去世后，为促进胸腺瘤的研究，其家族出资于2005年成立了全球首个关于胸腺肿瘤的基金会即FTCR（Foundation for Thymic Cancer Research）。2010年5月5日由美国NIH（National Institutes of Health）牵头，在FTCR的基础上于纽约成立了专业胸腺肿瘤学术组织，即ITMIG（International Thymic Malignacy Interest Group）。随后，日本也成立了相应的胸腺肿瘤学术组织即JART（Japanese Association for Research on the Thymus）。2012年6月由方文涛、陈克能等教授发起，在上海成立了中国胸腺肿瘤协作组（ChART），并与JART一同加入到ITMIG。

对胸腺瘤的深入研究，显然要寻找有效的方法来综合全世界的数据，这也是ITMIG（International Thymic Malignancy Interest Group）的主要目标。想要合作，就必须对该疾病使用相同的术语，但令人惊讶的是，现行的胸腺瘤实践中存在众多模糊定义和对相同概念有众多不同的解释，使得数据之间无法进行比较。因此，ITMIG联合全球的胸腺医务工作者对关键定义和方法共同讨论并达成共识，旨在促进胸腺瘤的研究。这项工作的结果收录在《胸部肿瘤杂志》（Journal of Thoracic Oncology）。ITMIG在建立这一广泛接受的共识时，首先由小组集中讨论提出初步建议，之后由扩大工作组（约80人组成）修订。然后将这些工作集中后于2010年11月15日-16日在耶鲁大学召开了为期2天的会议，对推荐的定义和方法进行讨论，并将结果分发给ITMIG的每位成员进一步讨论并反馈意见，最终修订版本经ITMIG批准并被采用。希望将来不管是单中心研究还是ITMIG的合作研究，都能采用这些标准。其中，因为胸腺瘤有一些不同于其它恶性肿瘤的特点，如除发病率低外，胸腺瘤还表现为惰性生物学行为，这对预后的临床评估有着诸多影响，因此，ITMIG特地撰写了“Standard Outcomes for Thymic Malignancies”，以供大家参考。

ITMIG共识中多数涉及患者的临床治疗，包括影像、活检、手术、放疗和化疗。对应用最广泛的Masaoka-Koga分期作了详细的细节规定。统一的定义将推动ITMIG的两个主要项目，即建立全球合作数据库和与IASLC的合作项目，为AJCC第8版肿瘤分期手册制订一个正式有效的胸腺瘤分期系统。必须强调的是，尽管要求统一的定义和方法，但并不意味它们是一成不变的，希望将来的研究能改善临床实践和共同使用的术语。同时鼓励对这些原则进行质疑和改进。

在ChART成立短短不到一年的时间里，已向ITMIG提供了信息较为完整的胸腺瘤病例资料近900例，占ITMIG目前所收集病例的四分之一，得到了ITMIG的充分肯定。2012年11月在日本福冈举办的第三届胸腺肿瘤会议上，ChART的4篇文章参加会议口头和壁报交流，并获得本次大会的Barbara Neibauer奖。同时积极参与了ITMIG目前正在筹划开展的两项前瞻性多中心国际合作课题，其一是前瞻性观察研究，其二是Ⅲ期胸腺瘤的全球多中心随机对照研究。

然而，与ITMIG一样，我国胸腺肿瘤学术界长期以来同样存在定义不规范，名词不统一等一系列问题，为此，经ChART研究，有必要就胸腺肿瘤的影像诊断规范、活检标本及切除标本的处理规范、临床及病理分期、预后评估标准、微创的标准用语及术式、放疗及化疗的定义及报告规范等问题做深入讨论，旨在进一步规范胸腺肿瘤的诊疗。首届论坛由北京大学肿瘤医院承办，邀请了影像学专家，病理学专家探讨胸腺肿瘤的诊断问题，同时邀请了外科专家，放疗科专家及内科专家，就治疗问题进行探讨，并将ITMIG提出的相关规定综合成册，供大家讨论应用。

2013年3月

